# Hybrid ray-tracing-QuaDRiGa/FDTD method for realistic 28 GHz exposure with 6G CF-MaMIMO in 3D outdoor environments

**DOI:** 10.1038/s44459-026-00031-4

**Published:** 2026-04-02

**Authors:** Robin Wydaeghe, Sergei Shikhantsov, Günter Vermeeren, Luc Martens, Emmeric Tanghe, Wout Joseph

**Affiliations:** Department of Information Technology, Ghent University/IMEC, Ghent, Belgium

**Keywords:** Engineering, Mathematics and computing

## Abstract

Amid worry for 5G and 6G, the layman’s question arises: “How much exposure do I *realistically* experience when I walk down the street?” Focusing on mmWave radio-frequency electromagnetic field exposure with Distributed Massive Multiple-Input Multiple-Output (DMaMIMO) technology, a new state-of-the-art (SOTA) numerical method is proposed to enable accurate exposure assessment almost anywhere on Earth. Google Earth 3D photorealistic tiles provide high-level-of-detail and high-coverage photogrammetry. We semantically classify the meshes with an SOTA deep learning model. The path of a pedestrian is first ray-traced at 28 GHz with either 6G DMaMIMO or realistically deployed 5G antenna systems as the transmitter. The large-scale fading parameters are extracted and form the input for the QuaDRiGa tool, which finely models the small-scale fading features of the channel along the full path with an omnidirectional User Equipment (UE) as receiver. The resulting channel is used in a hybridization procedure with a Huygens’ box that models the Electromagnetic Fields (EMFs) around the UE. The surface-absorbed power density (*S*_ab_) exposure metric is computed along the path using FDTD simulations of a realistic anatomical phantom. A case study in Helsinki finds that the cell-free MaMIMO free-space exposure range is 20 dB more uniform than collocated MaMIMO. A case study in New York City finds that users experience, on average, 20 dB higher values in exposure compared to non-users. The small-scale fading *hotspot* phenomenon in realistic environments is studied in detail, showing on average a 12 dB electric field increase w.r.t. the background and a specific shape with up to 3 sidelobes, which is characterized quantitatively. The *S*_ab_ is less than 1% of the ICNIRP guidelines during all simulations at realistic Tx powers.

## Introduction

The fifth (5G) and sixth (6G) generations of cellular networks address the ever-increasing demands in internet connectivity^1^. They feature order of magnitude increases in antenna density and frequency, aiming to increase the spectral efficiency and data rates^2^. Infrastructure for the 5G sub-6 GHz bands is becoming widespread, with some cellular providers even deploying millimeter wave (mmWave) Base Stations (BSs)^3^. Among the enabling technologies are (1) Massive Multiple-Input Multiple-Output (MaMIMO), antennas composed of a large number of elements^4^, and (2) the utilization of higher frequencies, such as mmWaves^5^. For the former, beamforming becomes possible by constructively interfering with each element’s wavefront through phase and amplitude modulation. Beamforming actively concentrates the emitted radiation optimally toward the User Equipment (UE), in contrast with fourth-generation BSs, which do not target active users. For the latter, higher bandwidths become available in less crowded frequency bands, such as FR2, which operates around 26–28 GHz. However, these higher frequencies incur greater path loss. One proposed solution is 6G Distributed Massive Multiple-Input Multiple-Output (DMaMIMO) BSs^6^. Elements are distributed, e.g., on a building facade, reducing the path length to the UE. Cell-Free MaMIMO (CF-MaMIMO) is a prospective variant of this technology, where a very large number of synchronized Access Points (APs) are distributed throughout a large geographical area^7,8^. Radio Stripes (RSs) are a prototype implementation of this paradigm^9^.

A portion of the population is concerned that these new technologies could lead to adverse health effects, sparking deployment stops, protests, and general worry^10,11^. Although deployed BSs always undergo stringent safety testing, research is needed to quantify the realistic Electromagnetic Fields (EMFs) from novel antenna systems in relation to the guidelines set by the International Commission on Non-Ionizing Radiation Protection (ICNIRP)^12^. A new surface-absorbed power density metric *S*_ab_ is introduced for frequencies above 6 GHz in the updated 2020 guidelines. Between the transmission of EMFs from the BS and the absorption in the human body, numerical dosimetry studies often focus on a specific part of the radio channel, making *worst-case* assumptions about other parts^13^. There is a gap in the current literature on *realistic* daily exposure from EMFs with comprehensive end-to-end simulations. For example^14,15^, introduce complex workflows to increase the realism of exposure estimation, but ultimately do not relate their findings to absorption values in humans. However, these findings would interest both concerned citizens and policy-makers due to 5G and 6G’s novelty, in particular as a result of MaMIMO technology. In addition, fully integrated simulations enable EMF-aware network planning. Here, the locations and powers of BSs in future networks can be realistically tuned before deployment. Finally, they enable the characterization of “hotspots”: wavelength-sized regions of increased EMF shaped by the precoding of BS transmission, the propagation environment, and the user^16–18^. Besides some general features, the detailed characteristics of hotspots in realistic environments are not known, especially at mmWave frequencies. Therefore, this work comprehensively studies hotspots in realistic environments at 28 GHz.

To model the exposure from these technologies, the *propagation step* (computing the incident fields in an environment) needs to be interfaced with the *exposure step* (computing the absorbed exposure on a human) in one integrated method. The MaMIMO BS estimates the channel between the receiver (Rx) and transmitter (Tx) using pilot waves. The transmitted symbols are then precoded in a process known as beamforming. For Line-Of-Sight (LOS) transmission scenarios, this causes (a) the direction of maximum gain to be aligned toward the Rx and (b) the individual elements’ phases of the Tx to align, causing constructive interference at the exact location of the UE’s antenna^19^. When several beams impinge on the UE, either from multipath components or from a DMaMIMO system, a wavelength-sized region of increased EMF is formed at the location of the UE. Under Maximum Ratio Transmission (MRT) precoding, the Signal-to-Noise Ratio (SNR) at Rx increases without bounds as a function of the number of antenna elements and channel diversity^4^. The maximum hotspot E-field also increases with the received signal, but the relationship is unclear. The objective of this study is to study the effects of these hotspots from MaMIMO BSs on human Radio-Frequency Electromagnetic Field (RF-EMF) exposure.

To inform the general public and policy-makers of realistic 5G EMF levels, and to enable EMF-aware network planning, integrated end-to-end methods should be able to map exposure almost anywhere in the world. Google recently introduced an API for their photorealistic meshes^20^. The digital twin of the world provides environments with full 3D Level of Detail (LoD), instead of 2D or 2.5D LoD, required for accurate mmWave radio channel modeling^21,22^. Conversely, the meshes are textured but not semantically labeled by Google. Recent advances in deep learning enable the semantic classification of these meshes to be performed with an accuracy of 93%^23^.

Hybrid Ray Tracing (RT)/Finite-Difference Time-Domain (FDTD) is an example of an integrated method^24^. The method was used to study hotspots at 3.5 GHz^16^. However, the first RT step is not appropriate to study exposure with high coverage in many different environments because RT is computationally intensive. Moreover, comprehensive data on the dielectric parameters and scattering of the materials is limited, especially at mmWave frequencies^25^. A new hybrid QUAsi Deterministic RadIo channel GenerAtor (QuaDRiGa)/FDTD method was proposed in^26^ based on the state-of-the-art (SOTA) QuaDRiGa^27^. QuaDRiGa renders the computations more efficient. However, the method is not suitable for site-specific simulations with high accuracy, as the Large-Scale Fading (LSF) does not align deterministically with the environment^17^. The computational requirements scale with the fourth power of frequency. Hence, there is a need to rethink the hybridization and exposure steps to obtain the exposure metrics efficiently.

To the best of the authors' knowledge, this work is novel in the following ways:

1. The first end-to-end method for exposure assessment at mmWaves. RT and QuaDRiGa simulations are leveraged efficiently, with a Huygens’ box to interface the incident EMFs to FDTD simulations.
2. Premier application of high-accuracy high-coverage photogrammetry in a scientific publication, in particular to compute the realistic exposure along any path. For the first time, a semantic classifier is applied to these meshes.
3. Comparison of collocated and cell-free MaMIMO in both user and non-user scenarios at 28 GHz.
4. Characterization of realistic hotspots at 28 GHz in realistic 3D environments. Their influence on the exposure metrics, the incident power density (*S*_inc_) and new surface-absorbed power density (*S*_ab_) for frequencies above 6 GHz, is examined.

## Methods

**Fig. 1:**
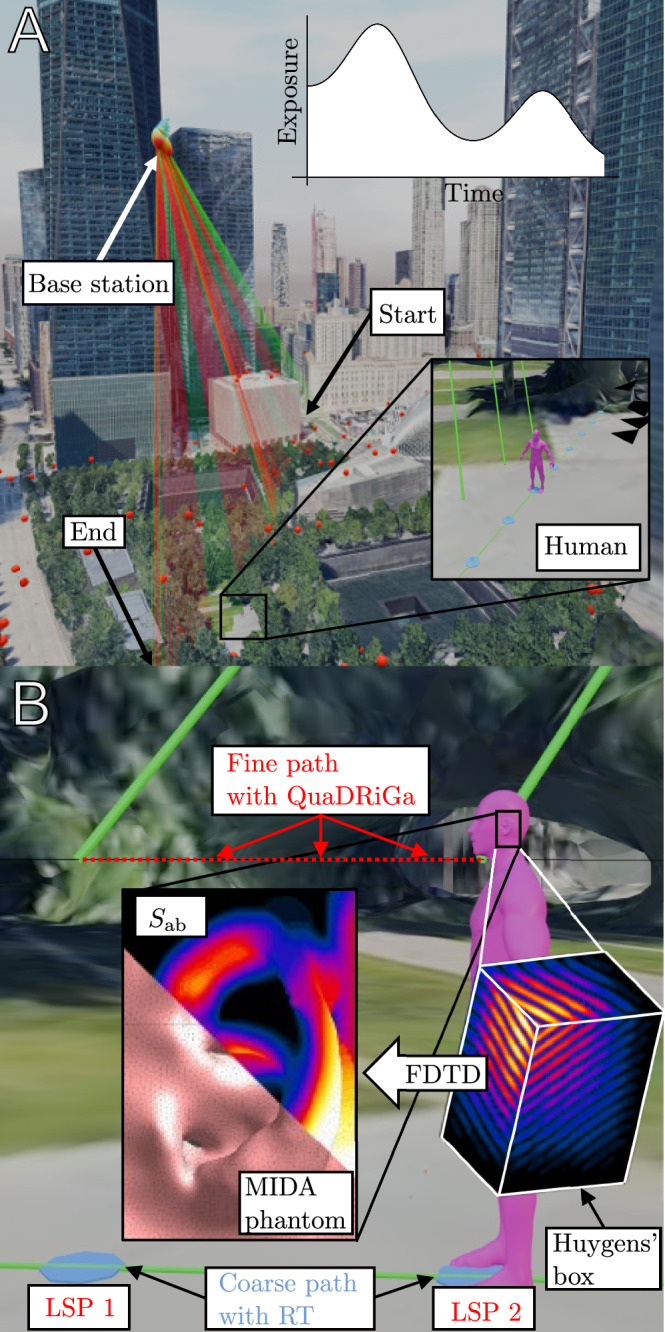
Illustration of the **a** configuration and propagation steps, and the **b** hybridization and exposure steps. **a** A walk (blue dots) in the WTC of NYC of a human (inset) is shown with the APs of a CF-MaMIMO system (red dots). The LOS/NLOS distinction from one specific AP is shown (green/red lines). A 3D realistic mesh from Google’s 3D Tiles API^48^ is retrieved to perform RT at 28 GHz on a coarse path. **b** A specific location is shown along the path. The blue dots denote the coarse points where RT is performed, from which LSPs are extracted. These form the input for the QuaDRiGa computations for the points in the fine path. The EMFs on a Huygens' are evaluated. These form the input for an FDTD simulation on the ear of the user, from which the *S*_ab_ exposure metric is extracted.

The integrated end-to-end method begins with a realistic walk of a user holding a smartphone and ends with the evaluation of the exposure metrics along this walk. The method chains 4 main steps in one pipeline. First, the configuration step, where the Rx, Tx, and environment are defined. Second, the propagation step, where the channel at the UE of the user is computed. Third, the hybridization step, where the EMFs impinging on the human body are computed. Fourth, the exposure step, where an FDTD and post-processing step return the exposure metric. Figure 1a illustrates the configuration and propagation steps, and Fig. 1b illustrates the hybridization and exposure steps.

### Configuration step

**Fig. 2:**
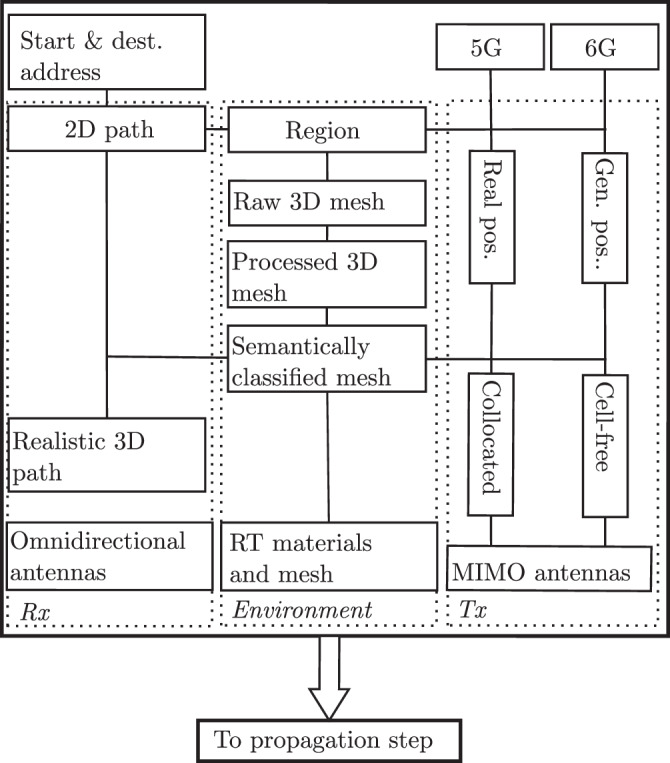
Flowchart of the configuration step.

A flowchart of the configuration step is detailed in Fig. 2. The only input is the start and destination addresses that a pedestrian traverses. This outputs a 2D path and region around it. The 2D path is processed to a sampled set of 3D points along the path in the environment that are omnidirectional antennas, representing the Rx. The region defines the environment, which is processed for use in RT simulations. The region also defines the locations of MIMO BSs, either collocated when studying 5G or cell-free when studying 6G.

#### Processed and classified environments

High-accuracy environments are essential for accurate ray-tracing simulations. In particular for mmWave frequencies, it is not sufficient to model building facades as flat surfaces^22^ or street canyons without cars or lampposts^28^. Foliage is rarely modeled with individual trees. The wavelength at 28 GHz is 10.7 mm. Therefore, centimeter-sized features significantly affect the multipath diversity of the channel. A greater number of NLOS paths can be utilized to beamform the signal toward the UE. The API for photorealistic 3D tiles^20^ is applicable to any location 3D-mapped by Google Earth. The reference in ref. ^29^ provides the first examination of its accuracy compared to established methods. The mesh includes vehicles, street furniture, individual trees, and building facade details, significantly improving the realism of the channel with clutter.

The input for the entire pipeline follows from the origin and destination of the user’s path. First, the quality of the mesh returned from the API is improved. Then, the mesh is split into 6 classes using a deep learning model^23^. Finally, the mesh is further simplified to be suitable for ray-tracing, and each class is assigned one suitable material. This three-step method is explained in more detail in the Supplementary Information.

#### Receiver path

The channel and exposure are computed along a path a user takes. The user’s path is composed of a series of waypoints forming the fastest path between the origin and destination addresses, as determined by the Google Maps Directions API^30^. These waypoints are then subsampled linearly. The Google API causes the user to walk realistic paths, as the waypoints are on walkable routes, such as sidewalks. To eliminate discontinuous changes in direction of travel, the user is modeled to always walk toward the point that is 2 m ahead on the straight path, subsampling the path every wavelength. The final path can clip with the environment, in which case the points are discarded. The elevation of the points follows the terrain. 1.5 m is added to this elevation to take the average height of the UE into account. The user walks at a constant speed of 1.4 m/s. Each point along the path is timestamped in order to average fields over time.

#### Antenna locations in a 5G network

The realistic locations of 5G BSs are not available on a global scale. One possible solution is open services that aggregate BS locations based on GPS location and signal strength through crowd-sourcing. For this work, the OpenCellID API^31^ provides the location of real cellular BSs with high coverage. Locations are determined using crowd-sourced data. Therefore, the accuracy depends on the number of measurements and the propagation model used to triangulate BSs' positions. Only the antenna density is representative of the physical twin. This is illustrated in Fig. 1a with red dots.

#### Antenna locations in a 6G network

A 6G CF-MaMIMO Access Point Network (APN) is a system that comprises a very large number of distributed APs which simultaneously serve a much smaller number of users over a wide area^7,8^. Here, APs are placed on the facades of buildings. First, building mesh faces whose normals have an azimuthal angle between 70° and 90° and with heights of 3 m above the ground are selected. Then, uniform Poisson disc sampling with a minimum distance of 10 m is performed using Bridson’s efficient algorithm^32^. 1.5 m above the nearest ground mesh, vertically polarized omnidirectional antennas model the UE (e.g., a smartphone) being held by the pedestrian.

### Propagation step

**Fig. 3:**
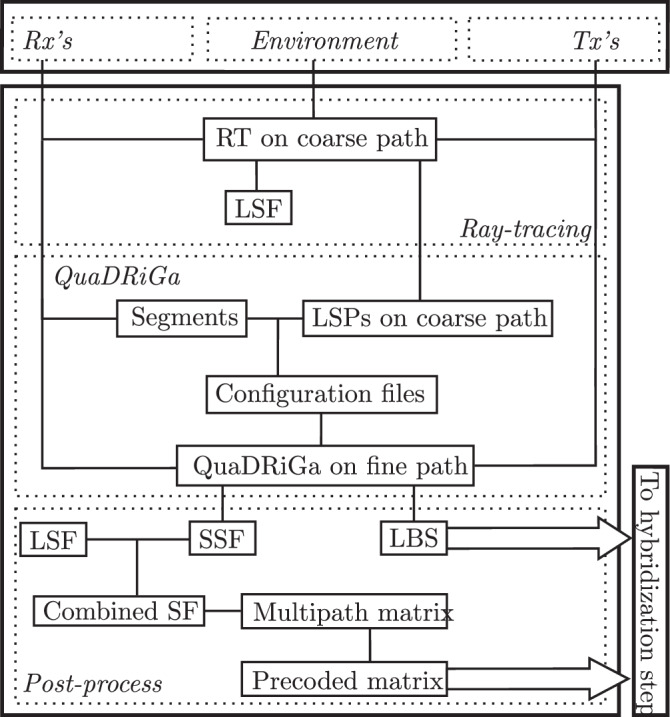
Flowchart of the propagation step.

**Fig. 4:**
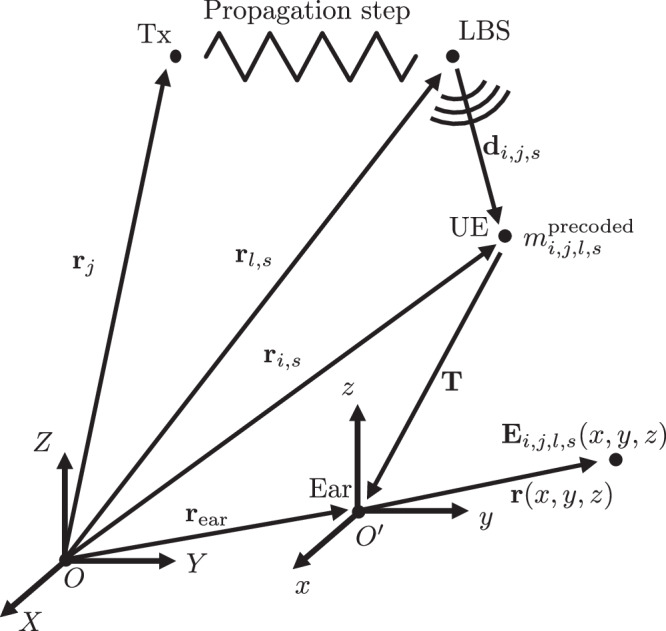
Overview of vectors related to the hybridization step.

A flowchart of the propagation step is detailed in Fig. 3. The upper box refers to the output of the configuration step in Fig. 2. In the central box, the propagation step combines RT and QuaDRiGa simulations for the LSF and Small-Scale Fading (SSF) modeling of the channel, respectively. These are post-processed and form the input for the hybridization step shown on the bottom right (Fig. 4).

In summary, from the configuration step, the Rxs along the path, the environment, and the Tx’s antenna elements are loaded in. This forms the input for RT simulations evaluated for all Rxs and Txs for every 1 m along the path. The output is the LSF channel and the 7 Large-Scale Parameters (LSPs) for each point along the coarse path. Using QuaDRiGa, the path is split into segments. Within each segment, the LSPs are statistically aggregated to form the input for the QuaDRiGa computation along the fine path. The output is the SSF channel and the Last-Bounce-Scatterers (LBSs). In a post-processing step, the former is combined with the LSF channel to obtain the *multipath matrix*, with entries for 4 dimensions: receivers *i*, antenna elements *j*, LBSs *l*, and distance snapshots *s*. Precoding is applied to the transmitted symbols. A *precoded multipath matrix* and the LBSs form the input for the hybridization step.

The propagation step involves simulating the wireless channel based on the configuration, defining all Txs, Rxs, and the 3D environment between them. Channel models can be broadly classified into deterministic and stochastic models. Based on the survey in ref. ^33^, the SOTA among these channel models is selected.

#### Ray tracing

RT is a deterministic channel modeling technique. In Shooting and Bouncing Rays (SBR) RT, rays shoot out from the Txs, propagate and scatter through the environment, and are collected at the Rxs. The advantage of RT is its potential to exactly model the underlying electromagnetic field problem in the high-frequency limit, especially suitable for 5G and 6G mmWave channel models. Therefore, RT enables site-specific simulations that can match the corresponding measurements^34^. An important disadvantage of the method is the need for an accurate description of EMF scattering properties for different materials in the scene^25^. The data on this remain sparse in the literature. Moreover, the LoD for the meshes must be in the order of the wavelength^22^. This requires a fine semantically classified environment at mmWaves frequencies. All these factors cause the accuracy of the Channel Impulse Response (CIR) to be insufficient for large-scale RT simulations. RT is also more computationally demanding compared to stochastic or map-based channel models. Derived parameters such as the LSPs and their correlation matrix agree more robustly with measurements than the CIR. RT simulations are done every meter along the path.

The commercial solver Wireless Insite^35^ is employed to perform the RT simulations. The Tx, Rx, and semantically classified environments are imported into the software. The six classes of materials are assigned homogeneous constitutive parameters (see Table S1 in the Supplementary Information). The SBR method is employed with 5 reflections, 1 diffraction, and 0 transmissions. The maximum angular separation between rays at the transmitter is 0.25^∘^. The radius of the collection sphere at the receiver is 20 cm. Diffuse scattering was not modeled. For each combination of *i*, *j*, and *s*, ${N}_{i,j,s}^{rays}$ rays are retained if their power is at least −250 dBm at the receiver. The result is cast in a *multipath matrix* **M**^RT^ with dimensions $N\times M\times ({\max} {N}^{rays}_{i,j,s})\times {N}_{snapshot}^{RT}$.

#### QuaDRiGa

The QuaDRiGa tool is used to generate realistic quasi-deterministic impulse responses from each AP to each receiver over time. QuaDRiGa is a quasi-deterministic channel model, but is classified as a Geometry-based Stochastic Channel Model (GSCM) with a large number of features^33^. Configuration files containing the stochastic information from measurement campaigns in LOS/NLOS urban/suburban environments are available in the software. Based on these, a spatially consistent stochastic parameter map of the LSPs is generated within each channel scenario^27^. Within segments along the path, stationary scattering clusters are generated around the receiver with properties that correspond to the parameter map. With the positions of the clusters known, a deterministic channel is obtained along the path of a moving user. This open-source model and its previous iterations have been validated for over two decades and comply with the 3GPP guidelines^36^. This tool was chosen as a component of our numerical pipeline for three reasons. First, QuaDRiGa provides realistic channels because the statistical parameters that generate parameter maps are derived from measurement campaigns^27,37^. In addition, version 2.0 features spatial consistency in the channels, which is important when modeling the resulting exposure along a path^38,39^. Finally, the channel generator is computationally more efficient than deterministic methods to model the large number of channels in CF-MaMIMO scenarios.

A number of LBS clusters, each with a number of subclusters, are generated around the user. The birth and death of clusters together with interpolations of their positions and powers also assure spatial consistency in the transitions between channel scenarios. Therefore, the hotspot shape and exposure metrics consistently conform to the path and environment variations. Subclusters improve the realism of the channel clutter effect by representing sub-resolution objects that accurately reproduce the LSPs from measurements. Computing the fields emitted from each subcluster is too computationally expensive. Instead, at most ${N}_{i,j,s}^{{\mathrm{subclusters}}}$ beams with the greatest power are selected for a combined channel. ${N}_{i,j,s}^{{\mathrm{subclusters}}}$ is chosen such that 99% of the power is accounted for in selected subclusters. Unselected subclusters have their total power (1%) spread uniformly over the selected ones. Multi-user scenarios can be implemented by defining an origin and destination address for each receiver and repeating the above steps. The QuaDRiGa software is modified to yield a multipath matrix **M**^QD^ with dimensions $N\times M\times ({\mathrm{max}}\,{N}_{i{,}j{,}s}^{{\mathrm{subclusters}}})\times {N}_{{\mathrm{snapshot}}}^{{\rm{Q}}{\rm{D}}}$, and the positions of the subclusters at each snapshot.

Hybrid QuaDRiGa/FDTD that is not based on RT uses the configuration files of the mmMAGIC model, valid for frequencies above 6 GHz, a UE height of 1.5 m, and a Tx height of 6–10 m^40^. For each point in the path, we manually assign a channel scenario based on the type of environment, e.g., dense urban, suburban, or rural. For each Tx, if the LOS connection is obstructed, the channel scenario is classified as NLOS and vice versa.

#### Ray tracing-based QuaDRiGa

Hybrid RT/FDTD was used for the first time at mmWaves in ref. ^24^. This enabled a realistic, site-specific evaluation of the exposure metrics on humans in a factory-of-the-future. Hybrid RT/FDTD is computationally challenging because the full EMFs for many directions of arrival (DoAs) need to be loaded to memory^41^. This was alleviated by introducing a Huygens’ box as an interface and applied to a small region in NYC in ref. ^17^. Despite improvements in efficiency, RT remains computationally demanding for environments larger than 100 m^2^.

QuaDRiGa was hybridized for the first time with the FDTD method in ref. ^26^. The propagation step could then be computed faster and with more APs than with hybrid RT/FDTD. This enables the computation of exposure on the city scale, with an order of magnitude more APs and users. However, results can only be interpreted statistically over a large number of samples. Due to the stochastic nature of GSCMs, the site-specific geometry is lost, with the exception of environment classification into LOS/NLOS and urban/suburban/rural.

This paper introduces for the first time, a new hybrid propagation model: *RT-based QuaDRiGa*. Falling under the map-based deterministic channel classification^33^, this model represents the SOTA in the wireless channel modeling literature by combining the benefits of both deterministic and stochastic models. Previous research has focused on substituting RT with QuaDRiGa using appropriate LSPs^42^. This work presents a method to retain the physical accuracy of RT while leveraging the speed of QuaDRiGa. Points every 1 m along the path of each user are chosen to evaluate the LSF variations in the environment. RT simulations compute the 8 LSPs used in QuaDRiGa^39^ for these points. QuaDRiGa splits the path for each receiver into segments. The length of segments is drawn from a normal distribution with a mean of 40 m, a standard deviation of 5 m, and a minimum/maximum of 30/70 m, respectively^40^. A new segment is also created when the receiver transitions between the LOS and NLOS states. Within these segments, the 7 LSPs (shadow fading is not considered) are aggregated statistically by computing the log-normal mean, standard deviation, decorrelation distances, and inter-parameter correlation matrices. These form the stochastic basis for the QuaDRiGa method. The data is collected in configuration files. These are generally used to characterize different radio environments classified by urbanicity. Then, QuaDRiGa computes the channel along the full path, capturing the SSF variations. For each Rx/Tx link, the LSF and SSF parts are combined.

Now, it is possible to construct the combined multipath matrix ${\bf{M}}\in {{\mathbb{C}}}^{N\times M\times L\times S}$ with $L={\mathrm{max}}\,{N}_{i{,}j{,}s}^{{\mathrm{subclusters}}}$ and $S={N}_{{\mathrm{snapshot}}}^{{\rm{Q}}{\rm{D}}}$. In general, the corresponding channel matrix **H**_*s*_ for snapshot *s* is the sum over either all subclusters in QuaDRiGa or all collected rays in RT in a link between receiver *i* and transmitter *j*:

1 $${\bf{H}}=\mathop{\sum }\limits_{l}{{\bf{M}}}_{l}.$$

**H**^QD^ and **H**^RT^ are interpolated and combined to form **H**^RT/QD^ (see Supplementary Information for the details). The final multipath matrix is obtained by:

2 $${{\bf{M}}}_{l}={{\bf{M}}}_{l}^{{\mathrm{QD}}}\odot | {{\bf{H}}}^{{\mathrm{RT}}/{\mathrm{QD}}}| \oslash | {{\bf{H}}}^{{\mathrm{QD}}}| ,$$

where ⊙ and ⊘ are the Hadamard element-wise multiplication and division operators. Using this rescaling, the more accurate amplitude information from RT is used, but the precise phase information from QuaDRiGa is retained. Note that the angle of arrival (AoA) information, including in NLOS conditions, still originates from the QuaDRiGa model.

#### Precoding

When computing the downlink exposure from a CF-MaMIMO 6G system^26^, assumed that every AP holds one antenna. However, these systems integrate multiple antenna elements within each AP. This is the case, e.g., in RSs. Due to the small wavelength at mid to upper mmWave bands, up to sixteen elements fit within the thin stripes, as is envisioned for 6G. Based on this, this paper presents results for a large number of APs of 4 × 4 patch antennas at 28 GHz, distributed in the environment. We consider a noiseless channel where each antenna element radiates with phases and amplitudes ${\boldsymbol{x}}\in {{\mathbb{C}}}^{M\times 1}$ and each UE receives a signal ${\boldsymbol{y}}\in {{\mathbb{C}}}^{N\times 1}$

3 $${\boldsymbol{y}}={\bf{H}}{\boldsymbol{x}}.$$

As precoding is performed similarly for each snapshot *s*, this index is omitted for clarity. Precoding is performed with MRT normalized to a power *P* using vector normalization. The precoding matrix **W** is^43^

4 $${\bf{W}}=\sqrt{\frac{P}{N}}\left[\frac{{{\bf{f}}}_{1}}{\parallel {{\bf{f}}}_{1}\parallel }\,\cdots \,\frac{{{\bf{f}}}_{N}}{\parallel {{\bf{f}}}_{N}\parallel }\right]\,,$$

where **f**_*i*_ are column vectors of the hermitian conjugate of the channel. As the antenna elements within one AP cannot cooperate with antenna elements from other APs, the precoding is performed in two steps.

In the first step, the powers and phases of each element in the 4 × 4 arrays are tuned to beamform toward the user. For an AP $J=1,\ldots ,{\mathcal{M}}$ that contains the antenna elements *j* with indices ${\mathcal{J}}(J)$, the entries of its associated precoding matrix are

5 $${w}_{j,i}^{J}=\left\{\begin{array}{lc}\frac{{h}_{i,j}^{* }}{\sqrt{N{\sum }_{j}| {h}_{i,j}{| }^{2}}} & \forall \,j\in {\mathcal{J}}(J)\\ 0 & \forall \,j\,\notin\, {\mathcal{J}}(J)\end{array}\right.$$

where *P* = 1 W. The phases and amplitudes of the antenna elements are independently added for each AP

6 $$\widehat{{\boldsymbol{x}}}=\mathop{\sum }\limits_{J}{\widehat{{\boldsymbol{x}}}}^{J}=\mathop{\sum }\limits_{J}\mathop{\sum }\limits_{i}{{\bf{w}}}_{i}^{J}{s}_{i}\,,$$

where ${\boldsymbol{s}}\in {{\mathbb{C}}}^{N\times 1}$ is the transmitted symbol. However, the phases and amplitudes are not final, as denoted by a hat, because there is no coordination between the APs yet. The received signal from AP *J* is

7 $${\widehat{{\boldsymbol{y}}}}^{J}={\bf{H}}{\widehat{{\boldsymbol{x}}}}^{J}\,,$$

or for each subcluster

8 $${\widehat{{\boldsymbol{y}}}}_{l}^{J}={\bf{M}}{\widehat{{\boldsymbol{x}}}}^{J}\,.$$

We can now define an intermediary multipath matrix $\widehat{{\bf{M}}}\,\in \,{{\mathbb{C}}}^{N\times {\mathcal{M}}\times L\times S}$ which embeds these phases and amplitudes in its matrix elements:

9 $${\widehat{M}}_{i,J,l,s}={\widehat{y}}_{i,J,l,s}\,.$$

In the second step, the phases and amplitudes of APs as a whole are tuned to beamform toward the user. The precoding matrix **W** of the entire APN is defined as in Eq. (4) but with

10 $${\bf{F}}={\widehat{{\bf{H}}}}^{H}\,,$$

where $\widehat{H}$ is the intermediary channel computed with Eq. (1). All exposure results are linear w.r.t. to the total power *P* allotted to the network in Eq. (4). The final phases and amplitudes of all antenna elements in the APN are

11 $${\boldsymbol{x}}=\mathop{\sum }\limits_{i}{{\bf{w}}}_{i}{s}_{i}\,.$$

The final *precoded multipath matrix* similarly embeds the transmitters’ phases and amplitudes with symbol ***s*** such that its entries are the received signal at the UEs:

12 $${m}_{i,j,l,s}^{{\mathrm{precoded}}}={\widehat{m}}_{i,j,l,s}\cdot {x}_{j,s}\,.$$

The precoded case will be compared with the *unprecoded* case. Here, *h*_*i*,*j*_ and *f*_*i*,*j*_ in Eqs. (5) and (10) are set to $\exp j{\phi }_{i,j}$ where *ϕ*_*i*,*j*_ are random numbers between $[0,2\pi )$. As the precoding acts on a completely different channel, the phases and amplitudes *x*^unprecoded^ will no longer cause beamforming toward the user. The final unprecoded multipath matrix is

13 $${m}_{i,j,l,s}^{{\mathrm{unprecoded}}}={\widehat{m}}_{i,j,l,s}\cdot {x}_{j,s}^{{\mathrm{unprecoded}}}\,,$$

where ${x}_{j,s}^{{\mathrm{unprecoded}}}$ arises from a normalized precoding matrix. MRT precoding cannot place a null on a non-user. A non-user can, however, experience increased exposure when entering the LSF hotspot from an active user. Hence, the case of unprecoded transmission, where beamforming is disabled, is comparable to a non-user scenario when aggregated over many samples.

### Hybridization step

The geometry of the hybridization step is shown in Fig. 4. A global coordinate system *O* defines the positions of an antenna element *j* in a MIMO BS (Tx), a QuaDRiGa LBS *l* at time snapshot *s*, the antenna of the UE held by user *i* at time snapshot *s*, and center of the evaluated exposure region (Ear) at time snapshot *s*. This center defines a new moving coordinate system ${O}^{{\prime} }$, parallel to *O* and translated by **r**_ear_. The final electric field *E*_*i*,*j*,*l*,*s*_(*x*, *y*, *z*) is defined in ${O}^{{\prime} }$ and emanates from the LBS (one of the scatterers in a QuaDRiGa cluster). The indices *i*, *j*, *l*, and *s* form a combination of Rx, Tx (element), subcluster LBS, and distance snapshot, respectively, for which the electric field is computed. The Huygens’ box function within the numerical pipeline is illustrated in Fig. 1b. A Huygens’ surface is used as an interface with the exposure step, requiring saving the fields only on the surface of the box^44^.

First, we describe the approach in free space. The “Exposure step” section will consider the case where the channel is altered because the impinging fields are scattered by the anatomical phantom. For a vertically polarized omnidirectional receiving antenna, the channel in Eq. (2) can be related to the electric field at the UE.

14 $${m}_{i,j,l,s}^{{\mathrm{precoded}}}=\frac{1}{{A}_{F}}{{\bf{E}}}_{i,j,l,s}(-{\bf{T}})\cdot {{\bf{e}}}_{\theta }({\theta }_{i,l,s},{\phi }_{i,l,s})\,,$$

where *A*_*F*_ is the antenna factor in m^−1^ of the omnidirectional antenna, *θ* and *ϕ* angles are the spherical coordinates of the impinging electric field **E**_*i*,*j*,*l*,*s*_ on the UE and **e**_*θ*_ is the unit vector along *θ*. This expression holds true at the antenna of the UE. The total fields are now computed on the bounding box around the location of the user’s right ear. Their coordinates ${\bf{r}}(x,y,z)$ are with respect to a new origin ${O}^{{\prime} }$ at **r**_ear_ = **T** + **r**_*i*_, where **T** is the translation between the UE’s antenna and the ear. In ${O}^{{\prime} }$, the electric field’s spatial component *ξ* in the far-field from a subcluster to ${\bf{r}}(x,y,z)$ behaves as

15 $$\xi (x,y,z)=A\frac{\exp (jk| {{\bf{d}}}_{i,l,s}+{\bf{T}}+{\bf{r}}(x,y,z)| )}{| {{\bf{d}}}_{i,l,s}+{\bf{T}}+{\bf{r}}(x,y,z)| }\,,$$

where **d**_*i*,*l*,*s*_ = **r**_*i*,*s*_ − **r**_*l*,*s*_ and *A* is its amplitude, normalized to unity at the UE

16 $$A=\exp (-jk| {{\bf{d}}}_{i,l,s}| )| {{\bf{d}}}_{i,l,s}| \,.$$

An expression can be constructed for the electric field around the UE:

17 $${{\bf{E}}}_{i,j,l,s}(x,y,z)={A}_{F}{m}_{i,j,l,s}^{{\mathrm{precoded}}}\xi (x,y,z){{\bf{e}}}_{\theta }({\theta }_{i,l,s},{\phi }_{i,l,s})\,.$$

By summing over all Txs and subclusters, the total EMFs are obtained

18 $$\begin{array}{rcl}{{\bf{E}}}_{i,s} & = & \mathop{\sum }\limits_{j,l}{{\bf{E}}}_{i,j,l,s}\,,\\ {{\bf{H}}}_{i,s} & = & \mathop{\sum }\limits_{j,l}\frac{1}{{Z}_{0}}\frac{{{\bf{d}}}_{i,l,s}}{| {{\bf{d}}}_{i,l,s}| }\times {{\bf{E}}}_{i,j,l,s}\,.\end{array}$$

These fields are evaluated on the sides of the Huygens’ box on the same Yee-grid as the FDTD simulation. Hence, the computational and memory requirements scale quadratically with frequency instead of cubically.

### Exposure step

#### Absorbed power density

The function of the exposure simulation within the numerical pipeline is illustrated in Fig. 1b. MRT precoding of the symbols causes the transmitted beam’s direction of maximal gain to be aligned toward **r**_*i*,*s*_, in particular for LOS and collocated antennas^41^. This is a *large-scale hotspot*, which can induce higher fields to nearby non-users. In addition, alignment of the amplitudes and phases from different beams causes an additional hotspot gain at **r**_*i*,*s*_ if **r** = −**T**. This is the *small-scale hotspot* and is studied in detail in this work. As reference quantities are determined in free-space receiving scenarios, the time-averaged incoming power density *S*_inc_ is readily available. However, to compute the basic quantities, an anatomical phantom is included in the FDTD simulations, which invalidates Eq. (14) and eliminates the small-scale hotspot effect. This user-induced coupling is included here based on ref. ^45^, but with the rays substituted by QuaDRiGa subclusters. The FDTD simulations themselves are performed similarly to those in ref. ^41^, but with efficiency and accuracy improvements. Both the details for the user-coupling and the FDTD simulations can be found in the Supplementary Information. The result is the absorbed power density along the path.

#### Hotspot characterization

In ref. ^17^, the shape of a hotspot was studied in an experimental setup. The hotspot features a large-scale hotspot around the receiver, a small-scale hotspot as a result of the interference pattern, prominent sidelobes, and spherical wavefronts. Similar results are seen at 3.5 GHz^16^. The aforementioned study validated the hybridization part of our tool because the experiment matched the simulation well.

Two methods to define the location of hotspots are examined. First, exactly where the UE is located, and second, where the fields are highest in a small region around the UE. At this location, three slices are taken normal to the *x*-, *y*-, and *z*-planes. A peak-finding algorithm detects whether a hotspot is present along each axis. We introduce three properties to characterize the hotspots:

1. The *dimensionality* of a hotspot is the number of axes on which a hotspot was found.
2. The Full Width at Half Maximum (FWHM) is the average of all detected FWHMs.
3. The Prominence-to-Peak (P/P) ratio provides a representation of the hotspot’s dynamic range, where a ratio of 1 is a peak flanked by fields equaling 0.

Two methods are proposed to average exposure metrics over time. In the first method, the exposure metrics *S*_inc_ and *S*_ab_ are considered for each time step in the walk. The average exposure metrics are then the average of all these over time. This is coined *exposure-wise* averaging, because the concept of “exposure at each time step” is correctly interpreted. These values correspond to the ICNIRP time-averaged exposure metrics for intervals of 6 min. In the second method, the average of the electric and magnetic fields over time is considered first. The average exposure metrics *S*_inc_ and *S*_ab_ are then computed for these average EMFs. This is coined *propagation-wise* averaging, because the concept of “average propagated EMFs” is correctly interpreted. These values provide insights into how interference typically forms the shape of a hotspot. Thus, the equations for incident power density are the following:

19 $$\begin{array}{rcl}{S}_{{\mathrm{inc}},{\mathrm{exposure}}-{\mathrm{wise}}}({\bf{r}}) & = & \langle | {\bf{E}}({\bf{r}})\times {\bf{H}}({\bf{r}})| \rangle \,,\\ {S}_{{\mathrm{inc}},{\mathrm{propagation}}-{\mathrm{wise}}}({\bf{r}}) & = & | \langle {\bf{E}}({\bf{r}})\rangle \times \langle {\bf{H}}({\bf{r}})\rangle | \,,\end{array}$$

where 〈 ⋅ 〉 is the average of a quantity with respect to time. Analogous formulas apply for *S*_ab_.

#### Exposure limits

In order to prevent excessive heating, the ICNIRP guidelines^12^ define limits for the reference and basic quantities. For the general public at 28 GHz, and for exposure durations longer than 6 min, the basic restriction is determined by *S*_ab_ averaged over 4 cm^2^ and 30 min, which should not exceed 20 W/m^2^. Likewise, the reference level for *S*_inc_ is 10 W/m^2^. It should be noted that far-field exposure, including that from MaMIMO technology, will typically induce highly non-uniform exposure patterns within intervals shorter than 6 min. In this case, the relevant exposure metric is integrated over time and compared with the brief local exposure limits^12^. However, these limits will always be less stringent than the corresponding 30-min time-averaged limits. Even though the results examine exposure over a walk of less than 6 min, we shall compare the instantaneous exposure metrics with the 30-min limits for reference and basic quantities, as it constitutes a worst-case scenario, and henceforth refer to them as the “exposure limits”.

Moreover, compliance with the reference levels is assumed to imply compliance with the basic restrictions. The relevant literature uses computational methods to assert this point under simplified and worst-case incidence assumptions. Therefore, it is sufficient to demonstrate compliance with only the reference levels. However, our tool computes both reference and basic quantities, providing a unique insight into the validity of this assumption under realistic incidence conditions, offering the possibility to relax or tighten the reference levels based on its conclusions.

## Results and discussion

Two case studies are examined, and the ray-tracing component is only introduced in the second. The first highlights the scalability of hybrid QuaDRiGa/FDTD, the second the accuracy of the entire pipeline. Then, the localized exposure around the UE is studied.

### Case study in Helsinki, Finland

**Fig. 5:**
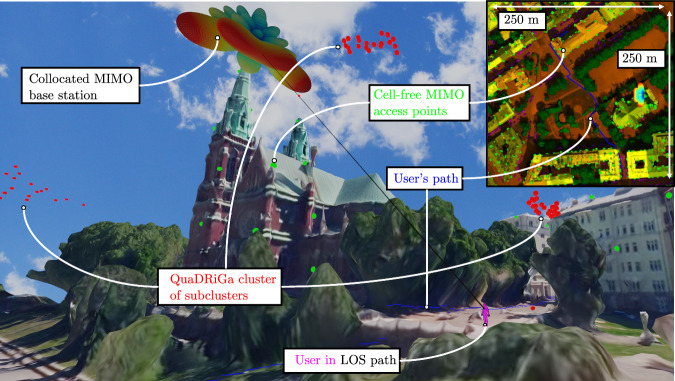
Configuration of the Helsinki case study. A CF-MaMIMO network (green) is simulated. Alternatively, a collocated MIMO BS beamforms toward the user (purple) at a snapshot in the LOS path (black). At this snapshot, the QuaDRiGa clusters, composed of subclusters (red), are shown (acting in free space). The inset depicts the user’s path (blue) walking from bottom to top.

In this case study, hybrid QuaDRiGa/FDTD is applied to a single-user scenario of a 6G cell-free massive MIMO network and compared with a 5G collocated MIMO antenna. Figure 5 shows the configuration of this case study. The signals are precoded toward a smartphone held in the hand of a pedestrian. The user walks the blue path shown in the inset of Fig. 5, near St. John’s Church in Helsinki. The total distance of the walk is 273 m. The pedestrian is in an urban environment in the first and last 50 m. In between, the user walks through the park of St. John’s church in central Helsinki. Due to the increased vegetation and absence of buildings, the environment in this section is suburban. An 8 × 8 collocated MIMO antenna is placed on the tower of the church, facing south and tilted down by 15^∘^.

The hybrid QuaDRiGa/FDTD method is applied with a carrier frequency of 28 GHz. The sampling rate is 53 Hz (such that there are 2.46 wavelengths between each snapshot), and the total number of distance snapshots (or evaluation points) is 9905. 335 distributed APs were generated in the 250 m × 250 m area around the path. The FDTD grid and Huygens’ surface have a resolution of 15 cells per wavelength, resulting in a grid of 302 × 230 × 323 cells. The average computation time for one snapshot of hybrid QuaDRiGa/FDTD is 160 s. In this case study, we choose **T** = −50 cm **e**_*x*_ + 50 cm **e**_*z*_, with the *x*-axis in the direction of travel and the *z*-axis in the vertical direction. This models holding a smartphone in front of the user’s torso. Thus, including head-coupling is not necessary as no hotspot gain is present. Note that the coupling is not necessary in free space when computing *S*_inc_ due to the experimental assessment procedure of reference quantities^12^.

**Fig. 6:**
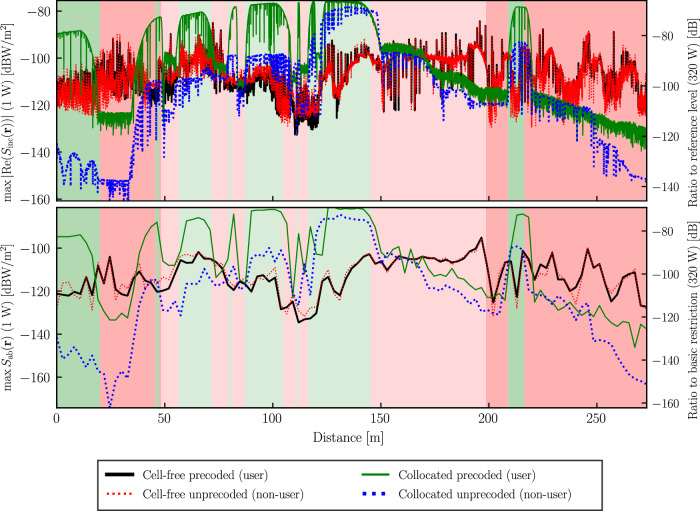
Reference (top) and basic (bottom) quantities as a function of distance along the path in Helsinki. Green or red patches indicate a LOS or NLOS state with the collocated antenna, respectively. The darker or lighter patches indicate an urban or suburban environment, respectively.

Figure 6 shows the reference and basic quantities as a function of distance along the path. The exposure is compared for precoded (user scenario) and unprecoded (non-user scenario) transmission, and compared for cell-free and collocated MaMIMO. The left axis expresses the exposure for normalized powers per antenna element. This means that the normalized exposure metric $\widehat{E}$ is

20 $$\widehat{E}=\frac{E}{{N}_{{\mathrm{AE}}}}\,,$$

where *E* is the exposure metric and *N*_AE_ equals 64 for the collocated antenna case or 335 for the distributed antenna case. The right axis expresses the ratio to the exposure limits for a typical total power of 320 W. This ratio *η,* therefore, equals

21 $$\eta =P\frac{\widehat{E}}{{E}_{{\mathrm{limit}}}}\,,$$

where *P* is the power (320 W), and *E*_limit_ is the maximum permissible level over a 30-min interval for the relevant exposure metric. At 28 GHz and for the general public, the reference level is 10 W/m^2^, and the basic restriction is 20 W/m^2^. The maximum of these ratios is at most 0.05% for a collocated BS precoding the channel in a suburban LOS propagation environment. The range between the minimal and maximal *S*_ab_ exposure is about 20 dB or 100 times greater with precoded transmission compared to unprecoded transmission, because the beams are steered in the direction of the user. This effect almost completely disappears for CF-MaMIMO, as can be seen in Fig. 6. At any time, only a few APs from different locations serve the user. The precoding gain is determined by the alignment of their phases and amplitudes, making beamforming impossible. The dynamic range of *S*_ab_ is also 20 dB smaller for the CF-MaMIMO network than for collocated MIMO. This is because the path loss is highest at the start and end of the path. In these urban regions, the user is also in NLOS due to the obstruction of the buildings. The cell-free APN causes a more uniform exposure along the path because, most of the time, at least one AP is in LOS with the user. From a network planning point of view, more power can be fed to a 6G CF-MaMIMO system compared to a 5G collocated one, while avoiding regions of excessive exposure. Furthermore, we observe the continuity (that is, no abrupt jumps) of the channel over time for LSF and report continuity for SSF. Finally, the Pearson correlation coefficients between the exposure quantities (in dB) are shown in Fig. S1 of the Supplementary Information. As was also found in ref. ^41^, the relationship between reference and basic quantities is strong, in this case approximately 0.60.

### Case study in New York City, USA

In this case study, RT-based hybrid QuaDRiGa/FDTD is applied to the urban scenario shown in Fig. 1a. After spending 10 s indoors, a smartphone user walks from the One WTC tower southward to Liberty Street in NYC, staying indoors for another 10 s. The user passes by an open area with foliage. Cellular APs from neighboring buildings radiate onto the user below the tree cover in either LOS or NLOS. The QuaDRiGa channel model distinguishes and interpolates between these in a continuous fashion. The total duration of the walk is 6 min. In this case study, we choose **T** = **0**, corresponding to the user holding a smartphone by the right ear. Thus, including head-coupling is necessary as a hotspot gain can be present.

The *user/non-user contrast* is defined as the average difference in signal gain between the two users walking 5 m from each other. The transmit symbol ***s*** = [1 0]^*T*^ is precoded such that the first user is a user and the second user is a non-user. Hence, a high user/non-user contrast shows functioning precoding and the extent of non-user exposure from a large-scale hotspot created by beamforming. When precoding is applied only on antenna elements within each AP (as in Eq. (5)), the user/non-user contrast increases by 6.01 dB along the walk. All APs beamform toward the user, but the closest APs do not necessarily emit most of the power. When precoding is applied only on the APs themselves, the user/non-user contrast increases by 4.74 dB. None of the APs beamform toward the user, but the nearest APs emit most power. When both precodings are combined (Eqs. (5) and (10)), the user/non-user contrast is 10.08 dB.

**Fig. 7:**
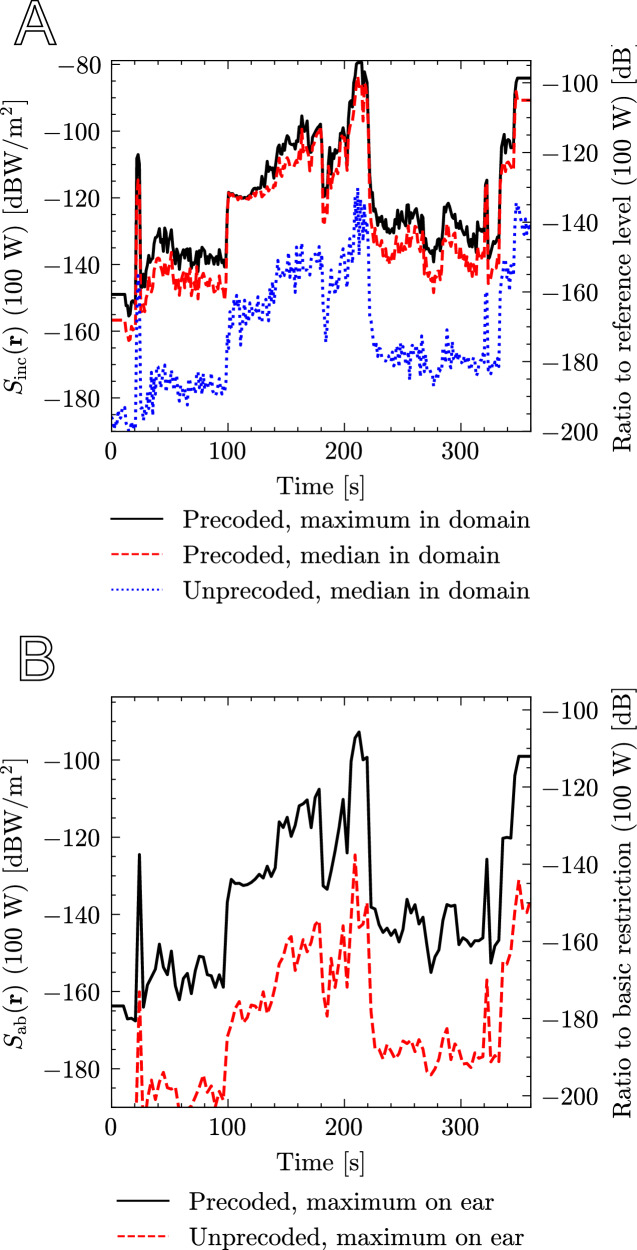
The reference (*S*_inc_) and basic (*S*_ab_) exposure quantities are plotted along the walk (left vertical axis). The right axis shows the ratio w.r.t. the ICNIRP limits for a 30-min averaging interval. The influence of the hotspot is shown in free space. **a** The reference quantity *S*_inc_ along the path. **b** The basic quantity *S*_ab_ along the path.

Figure 7 shows the exposure metrics *S*_inc_ and *S*_ab_ along the vertical axis on the left. The maximum *S*_inc_ is compared with the median *S*_inc_ in the computational domain to illustrate the hotspot gain. The precoded transmission case is compared with the unprecoded transmission case. The exposure is constant when the user is indoors. The exposure is continuous in time and consistent with the environment, e.g., the signal increases substantially when near APs and when the user is in LOS. The maximum exposure metric for *S*_inc_ and *S*_inc_ is −78.8 dBW/m^2^ and −92.8 dBW/m^2^, respectively, for 100 W total power in the CF-MaMIMO network. The ratio of the exposure metric divided by the relevant ICNIRP exposure limit is shown on the right vertical axis.

### Hotspots

Within a large-scale region exposed by the beams, hotspots can increase the aforementioned 10.08 dB gain locally, at or within several wavelengths of the receiver. A free-space domain of 4.76*λ* × 3.83*λ* × 6.63 *λ* (5.1 cm × 4.1 cm × 7.1 cm) is considered, fitting a human ear. The mean and standard deviation were computed based on 316 time steps along the walk. These results are shown in Table S2 of the Supplementary Information.

The means of the FWHM and P/P ratios indicate a size on the order of one wavelength (10.7 mm) and a visible peak, respectively. The distance from the center of the point with the highest field is 2.63 *λ* on average. However, the number of hotspots found along the path per *λ*^3^ is highest in the center. More than 50% of the hotspots are within 0.28 *λ* of the center, with many outliers. As is expected, the peak expresses high variability because of the log-normal distribution of received signals, as is typical in wireless channels. The standard deviations of the FWHM and P/P ratio are high, approximately 40% of their mean. When examining the field distribution along the walk step by step, the location and shape of the hotspot change frequently, but in a continuous fashion due to the time continuity of the propagation step. During 96% of the walk, the dimensionality is equal to 3, indicating a roughly ellipsoidal or spherical shape. The hotspots vanish or are reduced to a simple interference pattern when one or two strong LOS components are present, respectively. They also vanish when no precoding is applied.

**Fig. 8:**
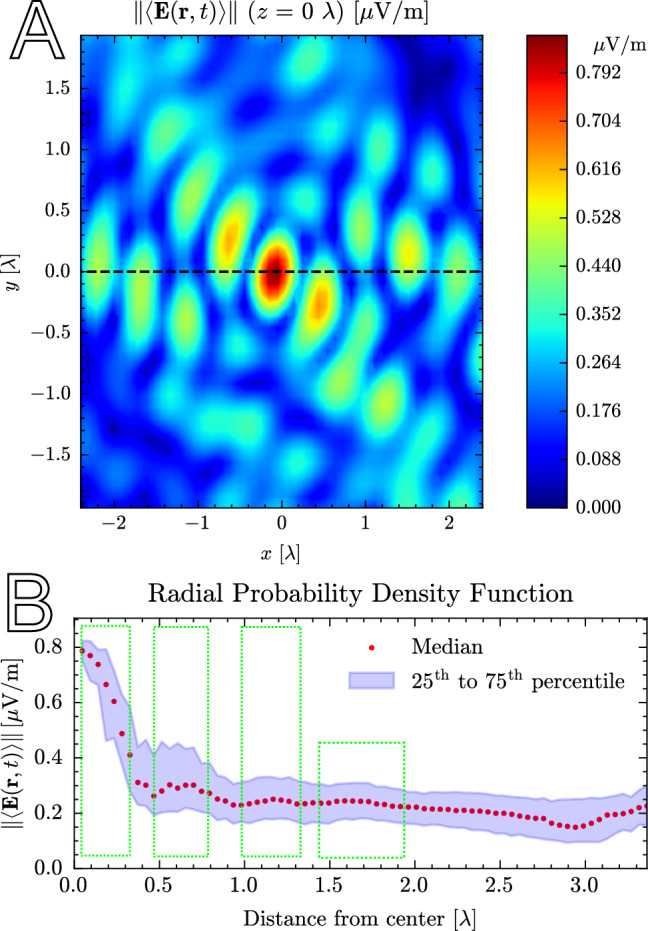
Visualization of a hotspot’s electric field when averaged propagation-wise (norm of the time-average). **a** A 2D slice through the focused UE. **b** The radial probability density function of the fields, showing the 25th, 50th, and 75th percentiles of all fields at distance (±0.15 mm). The green rectangles indicate the main, first, second, and (faint) third-order sidelobes of the hotspot.

At any specific time step, a hotspot can appear in a variety of shapes, sizes, and locations. However, a characteristic shape appears when the hotspot is averaged in time. ICNIRP also specifies that exposure metrics should be averaged over 6-min intervals. Figure 8a depicts a horizontal slice through the receiver, positioned in the center. In a first step, the electric field is examined because its norm features in Eq. (19) for propagation-wise *S*_inc_, and its field is maximized through precoding. The main peak is located in the center with an FWHM of 0.44 *λ*. The peak is flanked by troughs at 25% of its value, giving a ratio of 75% P/P (90% with the deepest troughs). A first-order sidelobe is observed at 0.61 *λ* with a peak value equal to 50% of the main peak. A second-order sidelobe is observed at 1.15 *λ*. Angular dependence is observed in the 2D slice, showing peaks and troughs for certain azimuthal angles. Figure 8b shows the radial probability density function of the field with respect to distance. For a distance *r* ± d*r* measured from the center of the FDTD domain, the field values are collected in a distribution. The graph displays the 25th, 50th, and 75th percentiles of this distribution as a function of *r*. The same main, first- and second-order sidelobes are observed (shown in green). A faint third-order is observed at 1.76 *λ*. This is fainter because the peaks and troughs are averaged out in the angular domain. As the distance approaches the edges of the domain, a constant field remains, which is 25% of the peak in the main lobe. Therefore, the small-scale hotspot increases the electric field by 12 dB on top of the large-scale beamforming gain. A higher number of antenna elements and APs increases this gain, in theory without bounds^46^. Within each sidelobe, peaks and troughs appear as a function of the azimuthal and elevation angles.

**Fig. 9:**
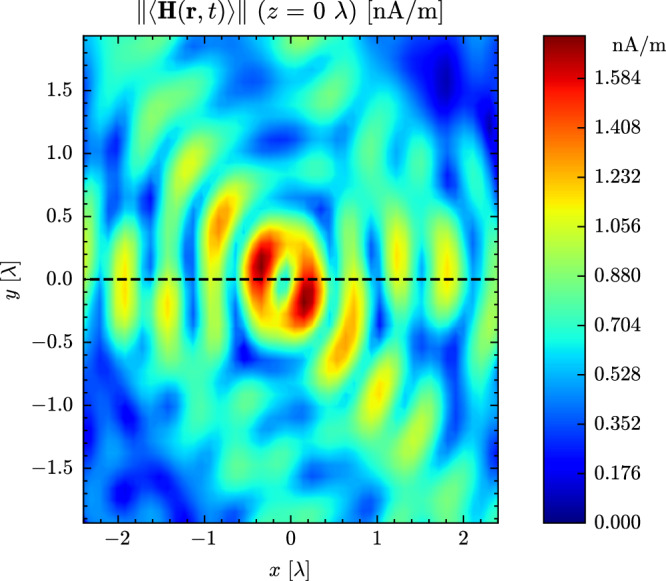
A 2D slice through the focused UE of a hotspot’s magnetic field when averaged propagation-wise (norm of the time-average).

**Fig. 10:**
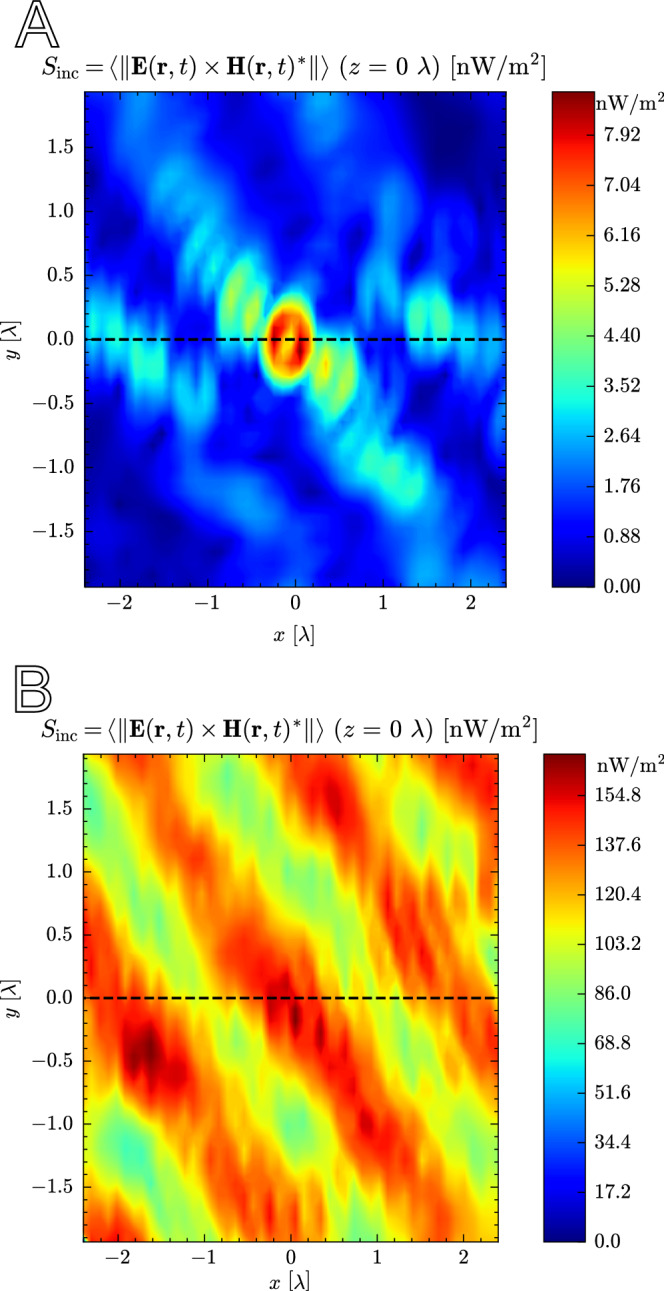
Comparison of the *S*_inc_ with the propagation- and exposure-wise averaging using 2D slices. The former averages the fields in time first, the latter computes the exposure first and then averages these in time. **a** Propagation-wise average of the hotspot (norm of the time-average). **b** Exposure-wise average of the hotspot (time-average of the norm).

The magnetic field of the hotspot behaves analogously to a typical interference pattern: when the electric field reaches a peak in space, the magnetic field reaches a trough, and vice versa, as can be seen in Fig. 9. Figure 10a shows *S*_inc_ for the 2D slice. The expression for *S*_inc_ combines the electric and magnetic fields. The hotspot also combines the features of the electric and magnetic-field hotspots. The small peak at the center is the combination of an electric field peak and a magnetic-field trough. This is flanked by higher peaks originating from the first-order magnetic-field sidelobes. The sidelobe series continues with alternating peaks from both fields. This can be seen more clearly in 1D slices (see Fig. S2 of the Supplementary Information) corresponding to the dashed line.

With propagation-wise averaging, positive EMF vector components at one time step can cancel out negative EMF vector components at another time step. However, from an exposure assessment point of view, both positive and negative field values can contribute to dielectric heating. The exposure-wise average of *S*_inc_ is visualized in Fig. 10b in the same 2D slice. A more chaotic interference pattern is seen with its highest peak not at the center. The FWHM is 0.92 *λ,* and the P/P ratio is 37% with the deepest troughs (see also Fig. S3 of the Supplementary Information). The pattern is overlaid on a constant envelope. This causes the first-order troughs to be 2.5 times less deep than with propagation-wise averaging.

This study has presented a comprehensive analysis of human exposure to EMFs in realistic environments using a novel RT-based hybrid QuaDRiGa/FDTD method. By integrating SOTA computational techniques and leveraging high-resolution 3D models from Google Earth, we have demonstrated an efficient and accurate approach to assessing exposure in 6G CF-MaMIMO networks operating at 28 GHz.

Key findings from case studies in Helsinki and NYC show the reference and basic exposure metrics along a realistic path, remaining within at most 1% of the ICNIRP guidelines limit for 30-min averaging intervals. The quasi-deterministic results indicate that the cell-free MaMIMO system provides a more uniform exposure compared to collocated MaMIMO, with a 20 dB reduction in path loss variability. Furthermore, the detailed hotspot analysis shows a 12 dB increase in electric field within these localized regions, emphasizing the importance of understanding small-scale exposure variations.

There are two key limitations of this study. First, ray-tracing complex scenes remains computationally intensive, especially for large urban environments with many reflective surfaces. Second, while ray-tracing provides deterministic amplitude information, the AoA information still originates from QuaDRiGa clusters.

The method enables (1) computing extensive exposure maps almost anywhere in the world and (2) studying a full digital twin of the entire pipeline, from Tx power control to exposure metric. These offer citizens and policy-makers insight into realistic EMF values, and network planners the ability to model exposure more accurately. Future work should focus on applying the method in different microenvironments and comparing it with measurements on city scales. Measurements can, e.g., be performed with a body-worn dosimeter and validated deterministically, provided a sufficiently accurate model of the Tx antennas is known. They can also be performed indoors, e.g., in a MaMIMO testbed^47^ to examine the shape of real hotspots, provided the Rx antenna does not distort the signal too much^26^. The 12 dB figure is expected to increase when this study is conducted for extremely massive MIMO systems. The method can be further improved with comprehensive data from realistic devices. The Tx and UE antenna patterns, as well as their locations and configurations, are the intellectual property of private companies. A wide outreach to industry from the scientific community in this field is important for realistic exposure assessment.

## Supplementary information

Supplementary Information

## Data Availability

The data that support the findings of this study are available from the corresponding author upon reasonable request.
