# Supplementary material for: Hybrid ray-tracing-QuaDRiGa/FDTD method for realistic 28 GHz exposure with 6G CF-MaMIMO in 3D outdoor environments
Source: NPJ Wirel Technol. 2026 Apr 2;2(1):13. doi: 10.1038/s44459-026-00031-4 (PMC13046467; doi:10.1038/s44459-026-00031-4)
Supplement: Supplementary file 1 — Supplementary Information [file 44459_2026_31_MOESM1_ESM.pdf]

Hybrid Ray-Tracing-QuaDRiGa/FDTD Method for Realistic 28 GHz Exposure with 6G CF-MaMIMO in 3D Outdoor Environments *Supplementary Information*

Robin Wydaeghe[1,*], Sergei Shikhantsov[1], Günter Vermeeren[1], Luc Martens[1], Emmeric Tanghe[1] and Wout Joseph[1].

I. Supplementary Results

A. Mesh processing in the configuration step

In the first step, we stream data at the highest Level of Detail (LoD) with a Blender renderer [1]. The result is a textured mesh. Common artifacts of photogrammetry data are present: non-manifold vertices and edges, duplicate vertices and faces, non-contiguous edges, and more. Moreover, some low-detail features contain an unnecessary amount of vertices, in particular at the seams of tiles. Therefore, the mesh quality is improved and its size is reduced.

1) The region along the path of the User Equipment (UE) is selected with a margin of 100 m.
2) The thin mesh is solidified by 50 cm.
3) The volume is voxelized with a resolution of 20 cm and adaptivity of 0 cm.
4) The initial texture is rebaked onto the new mesh using Meshlab.

In the second step, the mesh is semantically classified. This entails categorizing each face of the mesh in a number of pre-defined classes, such as buildings, ground, and vegetation. As our method aims to automatically compute the exposure for any location, classification must be performed automatically. With the accelerating capabilities of artificial intelligence, deep-learning semantic classifiers have become sufficiently reliable to perform this task for photogrammetry meshes. Both a clean mesh and the presence of a texture are essential for the performance of these models. The Semantic Urban Mesh Segmentation (SUMS) [2] deep-learning model features an overall accuracy of 93%. This model is pre-trained on a 4-km^2 area in central Helsinki, Finland. Environments with meshes and textures that do not resemble central Helsinki are expected to yield lower overall accuracy. Therefore, both Helsinki and New York City are picked for the studied case studies to examine the extent of overfitting the training set in the SUMS model. The Helsinki environment is lightly urbanized while the New York environment is heavily urbanized. Both feature an open area in the center of the walk.

[1]Department of Information Technology, Ghent University/IMEC, 9052 Ghent, Belgium.

*Corresponding author: Robin Wydaeghe (Robin.Wydaeghe@UGent.be).

This work is an extension of the conference submissions [?] and [?].

In the third step, the mesh was simplified by merging polygons lying on nearly the same plane, i.e., when the angle difference between them is less than 5°. This preserves small-scale features such as trees but simplifies large-scale features such as water. Hence, decimation reduces the face count of the considered environment and improves the efficiency of ray-tracing. For each semantic class, the most common material in that class is assigned to the corresponding digital twin, e.g., concrete for buildings.

B. Material parameters for the ray-tracing procedure

The constitutive parameters for the six types of materials are shown in Table S1.

TABLE S1: Constitutive Parameters for Different Classes of Materials in Ray Tracing at 28 GHz [3] [4]. Note that vegetation is not modeled as a stochastic scatterer.

**Material Class**	Relative permittivity ϵ_r	Conductivity σ (S/m)
**Buildings (concrete)**	5.24	0.6260
**Terrain (asphalt)**	5.7	$5 \cdot 10^{-4}$
**High Vegetation**	10	0.1
**Cars**	PEC[a]	PEC
**Water**	80	4
**Boat**	PEC	PEC

[a]Perfect Electric Conductor

C. Combination of the QuaDRiGa and ray-tracing channel matrices

Consider a segment along the walk starting and ending at a distance a and b, respectively. $\mathbf{H}_s^{\mathrm{QD}}$ are the channels obtained from QUAsi Deterministic RadIo channel GenerAtor (QuaDRiGa) simulations on distances $s\delta d$. $\mathbf{H}_n^{\mathrm{RT}}$ are the channels obtained from ray-tracing simulations on distances $n\Delta d = n(q\delta d)$, with $q = N_{\mathrm{snapshot}}^{\mathrm{QD}}/N_{\mathrm{snapshot}}^{\mathrm{RT}} \gg 1$. First, $\mathbf{H}^{\mathrm{QD}}$ is adjusted such that its start and end values align with $\mathbf{H}^{\mathrm{RT}}$ within the segment:

$$\widehat{\mathbf{H}}_s^{\mathrm{QD}} = \mathbf{H}_s^{\mathrm{QD}} + \frac{s\delta d - a}{b-a}\left(\mathbf{H}_{b/\Delta d}^{\mathrm{RT}} - \mathbf{H}_{b/\delta s}^{\mathrm{QD}}\right) - \frac{s\delta d - b}{b-a}\left(\mathbf{H}_{a/\Delta d}^{\mathrm{RT}} - \mathbf{H}_{a/\delta d}^{\mathrm{QD}}\right), \forall\, a \le s\delta d \le b\,.$$

This is repeated for all segments in the path. Second, the moving average of this adjusted function is subtracted from itself:

$$\widetilde{\mathbf{H}}_s^{\mathrm{QD}} = \widehat{\mathbf{H}}_s^{\mathrm{QD}} - \frac{1}{2u} \sum_{s'=s-u}^{s+u} \widehat{\mathbf{H}}_{s'}^{\mathrm{QD}}\,, \forall\, u \leq s \leq S - u\,,$$

where u is chosen here to be half of the Ray Tracing (RT) steps, $q/2$. The resulting $\widetilde{\mathbf{H}}$ captures the Small-Scale Fading (SSF) variations. Finally, a RT-based QuaDRiGa function $\mathbf{H}^{\mathrm{RT/QD}}$ is obtained by adding these SSF variations to the linear interpolation of $\mathbf{H}^{\mathrm{RT}}$ on the finer multiples of δd:

$$\mathbf{H}_s^{\mathrm{RT/QD}} = \mathbf{H}_{s,\mathrm{interp}}^{\mathrm{RT}} + \widetilde{\mathbf{H}}_s^{\mathrm{QD}}\,.$$

D. User-induced coupling of the channel

We opted to use a simple vertical electric edge source at 28 GHz, placed 1 cm orthogonally to the median plane from the tip of the right ear. A voltage of 1 V over the terminals is simulated using FDTD with the anatomical phantom. Based on [5], the reciprocity between transmitting and receiving [6] can be leveraged to compute the antenna pattern $A(\theta, \phi)$ directly using the far-field pattern of any UE antenna. However, this only holds for impinging plane waves, i.e., when the clusters are infinitely far. To prove this, consider a set of angles (θ_p, ϕ_p). For each, a cluster p is placed infinitely far with this orientation from the user in O'. Therefore, spherical wavefronts are neglected in the user-induced coupling. An impinging electric field from this cluster is defined such that the channel $m_{i,j,l,s}^{\mathrm{precoded}}$ equals 1 V at the UE in free space (fs):

$$1 = \frac{1}{A_F} \mathbf{E}_p^{\mathrm{fs}}\left(-\mathbf{T}\right) \cdot \mathbf{e}_\theta\left(\theta_p, \phi_p\right)\,.$$

Therefore, $\mathbf{E}_p^{\mathrm{fs}}$ is a plane wave with an amplitude of A_F and direction (θ_p, ϕ_p). By setting $\mathbf{E}_p^{\mathrm{fs}}(x, y, z)$ as source on the Huygens' box, an FDTD simulation is performed and the voltage V over the UE's antenna terminal is measured

$$A^\theta(\theta_p, \phi_p) \triangleq V\left(\mathbf{E}_p^{\mathrm{scattered}}\left(-\mathbf{T}\right)\right)\,.$$

The $\mathbf{E}_p^{\mathrm{fs}}$ field is scattered by the presence of the anatomical phantom to produce $\mathbf{E}_p^{\mathrm{scattered}}$ at the UE. Using this method, a vertically polarized antenna pattern $A^\theta(\theta, \phi)$ is retrieved. The uncoupled channel in free space can now be extended by this antenna pattern to obtain the coupled channel:

$$c_{i,j,l,s}^{\mathrm{coupled}} = A^\theta(\theta_{i,l,s}, \phi_{i,l,s}) c_{i,j,l,s}^{\mathrm{uncoupled}}\,,$$

where $c_{i,j,l,s}^{\mathrm{uncoupled}}$ refers to the final combined channel. Therefore, the precoding process should be executed only after this modification. When $\theta_{i,l,s}$ or $\phi_{i,l,s}$ does not correspond to one of the discrete angles (θ_p, ϕ_p), a bilinear interpolation is performed to estimate the antenna pattern. As the antenna pattern $A(\theta, \phi)$ needs to only be computed once for a specific exposure setup, this can be done as a preprocessing step.

TABLE S2: A comparison of hot-spot characteristics when defined to be at the receiver or at the point with the highest field near the receiver. The mean and standard deviation are given based on the S_{inc} metric in free space. In this table, d_{center} is the distance of the hot-spot from the center of the evaluation grid (at the UE), P/P ratio is the Peak-to-Prominence ratio of the hot-spot, Dim_3 is the percentage of hot-spots that have a dimensionality of 3.

	**At the receiver**	**At highest field**
d_{center} **[λ]**	0.0	2.79 ± 1.12
**Peak [nW/m²]**	0.19 ± 0.77	0.41 ± 1.65
**FWHM [λ]**	1.06 ± 0.41	0.88 ± 0.35
**P/P ratio [%]**	63 ± 26	57 ± 25
Dim_3 **[%]**	84.5	96.2

E. FDTD simulations

The surface currents from the Huygens' box form generalized total-field scattered-field sources in an Finite-Difference Time-Domain (FDTD) simulation that includes the Multimodal Imaging-Based Detailed Anatomical Model of the Human Head and Neck (MIDA) [7] as virtual phantom, featuring a spatial isotropic resolution of 500 µm. The surface needs to be gridded with a resolution of at least 10 cells per wavelength. The head is not oriented along the user's path. To reduce computational requirements, only a subset of the points along the path are computed. In FDTD simulations, the number of unknowns scale as f^3 and the simulation time as f [8]. However, all impulse responses for each Direction of Arrival (DoA) do not need to be kept in memory as in [9]. millimeter wave (mmWave) fifth (5G) and sixth (6G) operate at frequencies above 6 GHz, e.g. 28 GHz as used in this paper. The maximum time-averaged surface absorbed power density (S_{ab}) (in $\mathrm{W/m^2}$) on a 4-cm^2 surface is used to better account for the superficial nature of the exposure [10]. At 28 GHz, the penetration depth is approximately 0.92 mm $\approx \lambda/10$ [10], such that the field distribution cannot be resolved efficiently in FDTD. Therefore, a state of the art (SOTA) coated dielectric skin model is used on a homogeneous phantom [11], [12]. This exposure quantity is computed in a post-processing step with the skin of the virtual phantom [13]. Several steps were undertaken to increase the efficiency of the FDTD simulations. For example, simulations were run on a GPU-accelerated High-Performance Cluster, and the time-wise convergence was set to weak because the induced error falls within the margin of error already present in the pipeline.

II. Supplementary Discussion

A. Correlation matrix

The correlation matrix between the different exposure metrics is shown in Fig. S1. The table comparing the hot-spot characteristics is shown in Table S2. 1D-slices of the hot-spot's fields and incident power density are shown in Fig. S2 and S3, respectively.

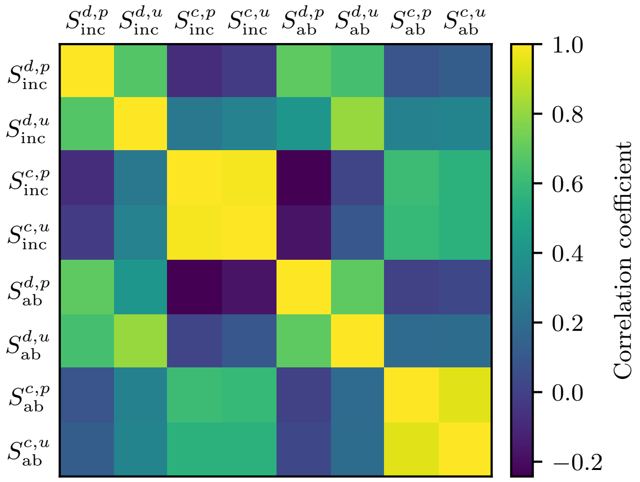

Fig. S1: Correlation matrix between the various exposure metrics shown for the Helsinki case study, using the Pearson coefficient of the values in dB along the walk. The superscripts c, d, p and u refer to collocated, distributed, precoded and unprecoded, respectively.

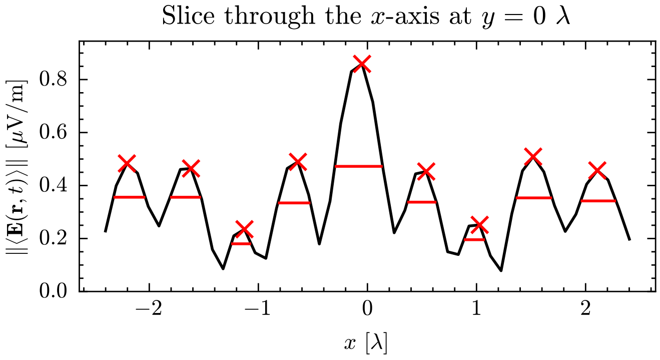

(a) A 1D slice of the electric field through the focused UE.

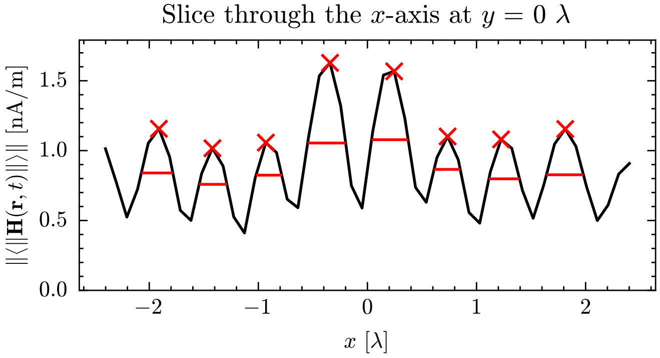

(b) A 1D slice of the magnetic field through the focused UE.

Fig. S2: Visualization of a hot-spot's electric and magnetic field when averaged propagation-wise (norm of the time-average).

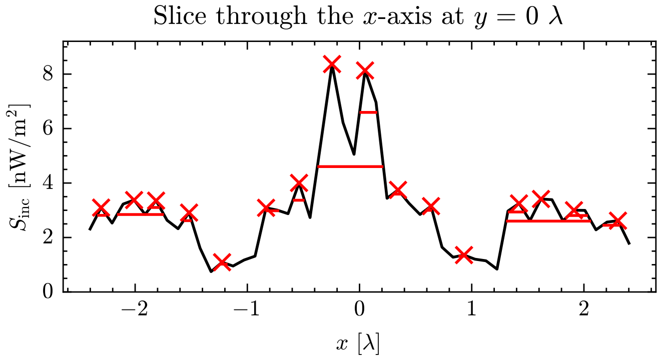

(a) Propagation-wise average of the hot-spot (norm of the time-average).

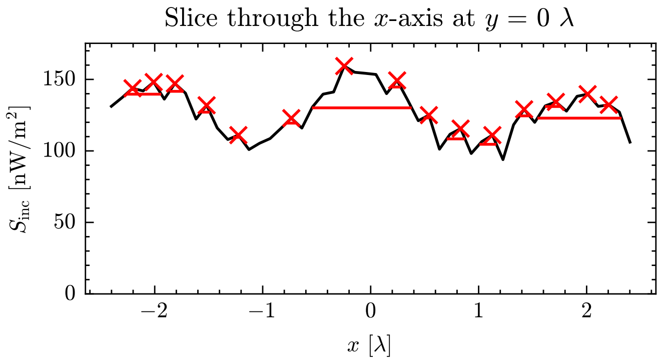

(b) Exposure-wise average of the hot-spot (time-average of the norm).

Fig. S3: Comparison of the S_{inc} with the propagation- and exposure-wise averaging using 1D slices. The former averages the fields in time first, the latter computes the exposure first and then averages these in time.

References

[1] vvoovv, "Blosm for Blender: OpenStreetMap, Google 3D cities, terrain," Github, 2023. [Online]. Available: https://github.com/vvoovv/blosm. [Accessed: Oct. 2024].

[2] W. Gao, L. Nan, B. Boom, and H. Ledoux, "SUM: A benchmark dataset of Semantic Urban Meshes,"

[3] International Telecommunications Union Radiocommunication Sector, "Effects of Building Materials and Structures on Radiowave Propagation Above About 100MHz," Recommendation P.2040, ITU-R, approved August 23, 2023. [Online]. Available: https://www.itu.int/rec/R-REC-P.2040/en. [Accessed: Oct., 2024].

[4] S. Shikhantsov *et al.*, "Ray-Tracing-Based Numerical Assessment of the Spatiotemporal Duty Cycle of 5G Massive MIMO in an Outdoor Urban Environment," *Appl. Sci.*, vol. 10, no. 21, p. 7631, Oct. 2020, doi: 10.3390/app10217631.

[5] S. Shikhantsov, "Massive MIMO Propagation Modeling With User-Induced Coupling Effects Using Ray-Tracing and FDTD," *IEEE J. Sel. Areas Commun.*, vol. 38, no. 9, pp. 1955-1963, Sept. 2020, doi: 10.1109/JSAC.2020.3000874.

[6] C. A. Balanis, *Antenna Theory: Analysis and Design*, 4th ed. Hoboken, NJ, USA: Wiley, 2016.

[7] M. I. Iacono et al., "MIDA: A Multimodal Imaging-Based Detailed Anatomical Model of the Human Head and Neck," Public Library of Science, vol. 10, no. 4, pp. 1-35, 2015, 10.1371/journal.pone.0124126.

[8] R. Wydaeghe *et al.*, "Realistic human exposure at 3.5 GHz and 28 GHz for distributed and collocated MaMIMO in indoor environments using hybrid ray-tracing and FDTD," *IEEE Access*, vol. 10, pp. 130996-131004, 2022, doi: 10.1109/ACCESS.2022.3227107.

[9] S. Shikhantsov *et al.*, "Hybrid Ray-Tracing/FDTD Method for Human Exposure Evaluation of a Massive MIMO Technology in an Industrial Indoor Environment," *IEEE Access*, vol. 7, pp. 21020-21031, Feb. 2019, doi: 10.1109/ACCESS.2019.2897921.

[10] International Commission on Non-Ionizing Radiation Protection, "Guidelines for limiting exposure to electromagnetic fields (100 KHz to 300 GHz)," *Health Phys.*, vol. 118, no. 5, pp. 483–524, 2020, doi: 10.1097/HP.0000000000001210.

[11] A. Christ, A. Aeschbacher, F. Rouholahnejad, T. Samaras, B. Tarigan, and N. Kuster, “Reflection Properties of the Human Skin From 40 to 110GHz: A Confirmation Study.” Bioelectromagnetics, 42: 562-574, Jul. 2021, doi: 10.1002/bem.22362.

[12] *Sim4Life Application and Support Team*, *Sim4Life Reference Guide*, Release 7.2, Zurich, Switzerland: ZMT Zurich MedTech AG, Dec. 2022.

[13] IEC/IEEE International Standard - Assessment of power density of human exposure to radio frequency fields from wireless devices in close proximity to the head and body (frequency range of 6 GHz to 300 GHz)–Part 2: Computational procedure, IEEE/IEC 63195-2-2022, May 2022.
